# Do laying hens eat and forage in excreta from other hens?

**DOI:** 10.1017/S1751731118001143

**Published:** 2018-05-28

**Authors:** C. G. von Waldburg-Zeil, N. van Staaveren, A. Harlander-Matauschek

**Affiliations:** Department of Animal Biosciences, University of Guelph, 50 Stone Road E, Guelph, Ontario, Canada N1G 2W1

**Keywords:** coprophagy, faeces, feeding preference, birds, liver enzymes

## Abstract

Worldwide, farm animals are kept on litter or foraging substrate that becomes increasingly soiled throughout the production cycle. For animals like laying hens, this means that it is likely they would scratch, forage and consume portions of excreta found in the litter or foraging substrate. However, no study has investigated the relative preference of laying hens for foraging and consumption of feed mixed with different percentages of excreta. A total of 48 White Leghorn laying hens of two strains, a commercial strain (Lohmann LSL-Lite (LSL), *n*=24) and UCD-003 strain (susceptible to liver damage, *n*=24), were individually housed and given access to feed mixed with increasing percentages of hen excreta (0%, 33%, 66% and 100% excreta diets) and corn as a luxury food reward (four corn kernels per diet daily). The amount of substrate and number of corn kernels consumed from each diet was recorded for a period of 3 weeks. Both LSL and UCD-003 hens preferred to consume and forage in diets with 0% excreta, followed by 33% and finally diets containing 66% and 100% excreta. Despite the presence of excreta-free diets, birds consumed on average 61.3 g per day of the diets containing excreta. Neither physical health, measured by plasma enzyme activity levels, nor cognitive differences, assessed by recalling a visual discrimination task, was associated with relative feeding or foraging preference. In conclusion, this study demonstrated a clear preference for feeding and foraging on substrate without excreta in laying hens. However, considering the amount of excreta diets consumed, further studies are needed to understand the causes and consequences of excreta consumption on physiological and psychological functioning, and how this information can be used to allow adjustments in the management of foraging substrates in farmed birds.

## Implications

This research provides new insight into feeding and foraging behaviour of laying hens, clearly demonstrating that they prefer to feed and forage in diets free of excreta. Significantly lower quantities were consumed from diets with 33% or higher percentages of excreta; however, hens still consumed a considerable amount of substrate from excreta diets. The causes and consequences of excreta consumption in laying hens need to be further investigated before potential adjustments can be made to the management of foraging substrates in farmed birds.

## Introduction

Farm animal welfare is receiving increasing attention worldwide with implications for farmers, retailers and policy makers (Horgan and Gavinelli, 2006). The concept of animal welfare encompasses three perspectives, which are based on biological functioning, subjective experiences and naturalistic living (Fraser *et al*., 1997). Society has become intently focused with the latter as a key value of animal welfare, expressing growing concerns about unnatural, monotonous environments where animals have few opportunities to perform natural behaviours (Spooner *et al*., 2014; Ventura *et al*., 2016). In natural environments, animals forage to select a mixed and diverse diet to meet their nutritional needs and many farm animals (e.g. cows, chickens, pigs) are highly motivated to perform this foraging behaviour (Bailey, 1995; Provenza, 1995; Bracke and Hopster, 2006; Weeks and Nicol, 2006; van de Weerd and Day, 2009). However, farming environments are often non-naturalistic and monotonous, limiting animals’ foraging opportunities (Manteca *et al*., 2008; Mellor, 2017). Consequently, farming environments were created that include litter-based systems that offer the opportunity for expression of natural behaviours such as scratching, chewing and possibly eating of the substrate. However, in these systems there is an increased likelihood of animals foraging on and ingesting excreta, especially when this litter substrate is not replaced during production.

It is well documented that many domesticated livestock species selectively forage away from excreta, reducing disease transfer and parasite loads in these animals (Forbes and Kyriazakis, 1995). In addition, separate functional areas, for example resting, feeding and fouling are considered important in the design of housing for many farm species (Mishra *et al*., 2005; Fernández *et al*., 2006; Buijs *et al*., 2010; Vermeer *et al*., 2014). As such, by providing litter that becomes increasingly soiled throughout the animal’s life, there is potential for unknown impacts on farm animal welfare. Nevertheless, several avian and mammalian species occasionally consume small amounts of excreta, which is suggested to provide nutritional benefits (Hörnicke and Björnhag, 1980; Negro *et al*., 2002; Horgan and Berrow, 2004; Shimada, 2012). However, the occurrence and importance of this excreta-consuming behaviour (i.e. coprophagy) when animals are kept on litter mixed with excreta are largely unknown. Interestingly, in humans the consumption of excreta has been linked to particular metabolic diseases and is associated with lower cognitive functioning (Ali, 2001). However, to the authors’ knowledge, it has not been investigated whether the ingestion of excreta is associated with metabolic diseases and/or lower cognitive functioning in domestic animals.

Using laying hens as an example, this study tried to address this knowledge gap of whether or not coprophagy occurs in domestic farm animals. Laying hens are highly motivated to forage (Weeks and Nicol, 2006; Widowski *et al*., 2013) and changes in laying hen housing have been implemented to transition from conventional cages (‘unnatural’ environment) to non-cage systems (‘natural’ litter-based environment) affecting the management and welfare of laying hens across the world (European Commission, 1999; National Farm Animal Care Council (NFACC), 2017). While legislation and/or recommendations state that good quality foraging substrate should be provided, no specific recommendations are given on the management of this material. Previous research investigating the outcomes of litter provision has largely focused on positive effects, such as allowing the expression of natural behaviours, or the more negative effects, such as egg contamination, foot disorders and animal or worker health in relation to ammonia or dust levels (reviewed by Rodenburg *et al*., 2005). However, the issue of whether laying hens consume the excreta in the litter has not been investigated, even though it is known that excreta in the litter can increase greatly over the course of the laying period (Groot Koerkamp, 1994).

In order to determine the relative preference of adult laying hens for commercial feed/forage mixed with various percentages of excreta from conspecifics, a preference test assessing whether hens ingest excreta was conducted. Second, the motivation to search for and ingest a luxury food reward (i.e. corn kernels) in these diets was measured. Finally, it was investigated whether the ingestion of excreta could be linked to metabolic disorders and/or cognitive abilities by using a commercial strain of single comb white leghorn laying hens (Lohmann LSL-Lite (LSL)) and an experimental strain (fatty liver hemorrhagic syndrome susceptible strain (UCD-003)) as a metabolic disorder model and determining whether these strains differed in their feeding preference and ability to solve a visual discrimination task. A primary motivation for this study was to improve understanding of feeding behaviour of laying hens, and may be applied in the management of foraging substrate for the improvement of laying hen welfare.

## Material and methods

### Animals and housing

This study was approved by the University of Guelph Animal Care Committee (Animal Utilization Protocol Number 3169). Before the choice feeding experiment, laying hens were kept in groups of several hundred birds on a combination of deep litter (pine shaving-excreta mix) and slatted floor housing. A total of 48 adult laying hens (71 weeks of age) comprised of two different strains (LSL, UCD-003; *n*=24 for each strain) were individually housed in commercial cages (61×62×53.5 cm; FDI Cage Systems, Mitchell, Ontario, Canada). Each cage was furnished with a perch (30×2.54 cm) and scratch mat (20×15 cm) and equipped with nipple drinkers and a feeding trough running lengthwise in front of the cage. Opaque plastic barriers were placed floor-to-ceiling on the front half of the adjacent cage walls (25×40 cm) to avoid social learning influencing feed preferences. Lighting and temperature schedules were provided as recommended in management guidelines.

### Experimental design and diet treatments

Data collection began after 1 week of habituation to the experimental set-up (including housing and diets). Hens were presented with four treatment diets at different feed : excreta ratios, 1: 0% excreta, 2: 33% excreta, 3: 66% excreta, and 4: 100% excreta. Feed was a standard diet provided by the Arkell research station (CP – 18%; calcium (Ca) – 4.2%; available phosphorus (P) – 0.44%; sodium (Na) – 0.18%; metabolizable energy (ME) – 2 900 kcal/kg; lysine – 0.89%; methionine – 0.38%). Fresh excreta (moisture – 96.3%; pH – 6.1; nitrogen (N) – 1.77%; ammonia (NH_3_) – 3503.05 ppm; SGS, AgriFood Laboratory, Guelph, Canada) were collected daily from non-experimental adult laying hens receiving the same feed and kept under the same housing conditions. Fresh excreta were mixed in with the feed manually to ensure homogeneity. Four equally sized aluminium containers (14.5×4.8×8.4 cm) were filled with each diet at the same volume but slightly different weight (134.3±10.16 g), and were placed in the feed trough at the front of the cage with plastic dividers (height: 12.7 cm) between them to avoid diet spillage and/or contamination. The containers were emptied and refilled on a daily basis (0830 to 1200 h). In addition, four kernels of corn (Great Value Golden Sweet Whole Kernel Corn, Walmart #00924892) were placed in each container to promote foraging. The order of the containers filled with the four different diets was systematically varied throughout the experiment which was conducted over 21 days. Consumption of each diet was recorded daily through back weighing of each container using a digital scale (NAGATA Model FAT-12, TNN, TW) and the number of corn kernels consumed from each container was recorded daily. The corn kernels were not included in the weight of the containers during recording. On the first day, corn kernels were added to the diet containers after initial weighing. The following days, corn kernels were located and removed from the containers to determine the number of corn kernels consumed, after which the diet containers were weighed to assess the amount of substrate consumed.

In addition, BW was recorded for all hens and blood samples collected (1000 to 1030) on day 1 and day 21 of the experiment to measure plasma enzyme activities. Enzyme activity levels of alanine aminotransferase (ALT) indicating non-specific cell damage (Hochleithner, 1994), aspartate aminotransferase (AST) indicating liver damage when values are above 230 U/l (Hochleithner, 1994), and gamma-glutamyl transferase (GGT) indicating bird liver and biliary compromises when outside of the normal range of 0 to 10 U/l (Harr, 2006) were determined to assess whether the UCD-003 hens indeed differed in their liver health compared with LSL hens (Diaz *et al*., 1999). Venous blood (1.5 ml) was drawn from the wing and stored into EDTA coated tubes and centrifuged for plasma collection (3000×**g** at 4°C for 10 min). Plasma was frozen at −20°C and submitted to the Animal Health Laboratory (University of Guelph) for analysis of AST, ALT and GGT activity levels using the Roche Cobas C ASTL kit ID 0-494, C ALTL kit ID 0-495 and Roche Cobas C GGT-2 kit version 2 (Roche Diagnostics, Indianapolis, IN, USA), respectively.

### Visual discrimination task

Cognitive function was evaluated in a random subsample of 12 laying hens (six birds per strain) to assess possible differences between LSL hens and UCD-003 hens. A two-choice discrimination task in a Y-maze was used to assess the rate of acquisition of a simple visual discrimination and memory task. Birds were habituated to the maze from day 1 of the experiment for three consecutive days by allowing birds to explore the maze freely during a 5-min period.

#### Apparatus

**Figure 1:**
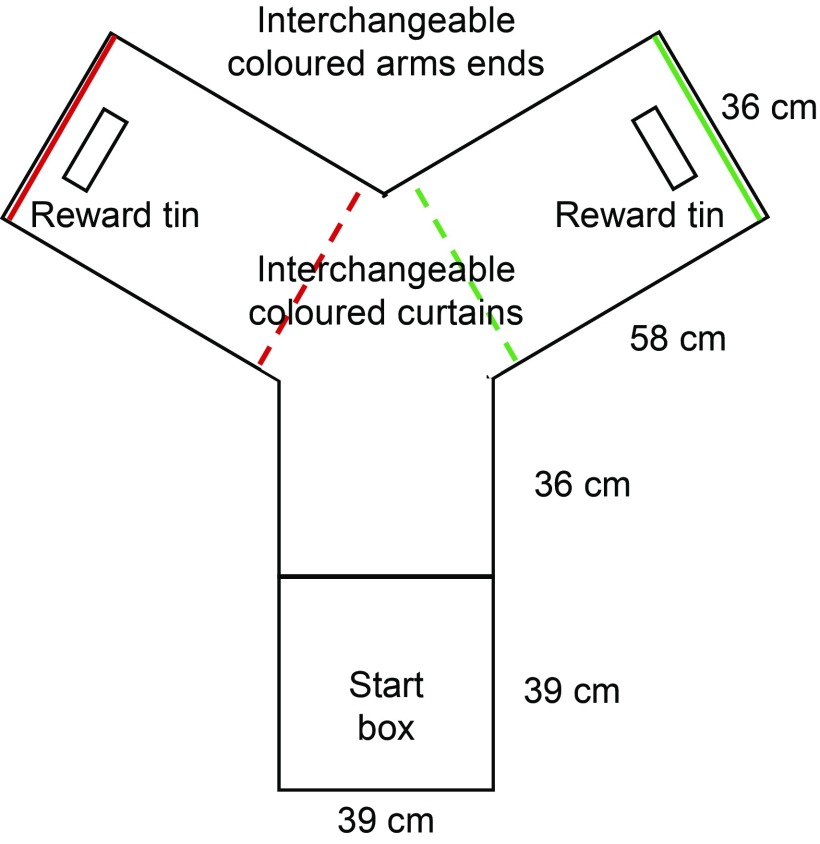
(colour online) Y-maze construction used by laying hens during visual discrimination task.

The Y-maze was constructed of white poster board (height: 61 cm) and a black Styrofoam floor (Figure 1). It compromised a start box (39×39 cm) with a connecting alleyway (36×38 cm) which split into two arms (58×38 cm) arranged in a Y-shape with a goal area in each arm. The entrance of the arms was colour coded by interchangeable cotton cloth strip curtains (red and green). Rectangular containers (14.5×4.8×8.4 cm) containing corn kernels were placed in the goal area in each arm. However, the corn in the container at the non-reward side was covered with a perforated plastic screen to prevent birds from obtaining the reward and to control for olfactory cues.

#### Training and testing

Half of the hens were trained to find the corn kernels at the red or green arm of the maze, respectively. The red and green arm were systematically alternated to control for side-bias. Training ran for 7 days until each bird had passed the learning criterion. The learning criterion was defined as 5 out of 6 consecutive correct runs. If a bird entered the non-reward arm or failed to enter either arm within 90 s, the run was recorded as incorrect. A run was considered as correct when the hen completely passed through the curtain and ate the food reward (10 s access to the food reward) at the end of the arm. Each hen completed a maximum of 10 runs per day. The number of runs needed to reach the learning criterion was recorded. Testing to see how well birds had memorized the task began on day 21 of the experiment. The correct side of the colour stimulus was systematically varied for each run. The same procedure was followed as during training and the number of runs needed to reach the learning criterion (5 out of 6 consecutive correct runs) was recorded.

### Statistical analysis

All statistical procedures were conducted using SAS V9.4 (SAS Institute Inc., Cary, NC, USA). The assumptions of normally distributed residuals and homogeneity of variance were examined graphically with the use of QQ plots. Statistical significance was considered at *P*<0.05. Values are presented as LS means±SE, unless stated otherwise.

The average percentage of substrate (feed and/or excreta) and total number of corn kernels consumed per diet was calculated per week as an indicator of preference. Generalized linear mixed models (PROC GLIMMIX) were used to determine the effect of diet (0%, 33%, 66%, 100% excreta), strain (LSL, UCD-003) and their interaction on the percentage of substrate or number of corn kernels consumed, with bird included as the repeated subject. A binomial distribution was used for analysing the proportion of corn kernels consumed by expressing the total number of corn kernels consumed out of the maximum number of corn kernels present per diet per week. A Tukey–Kramer adjustment was used to account for multiple comparisons. The activity levels of AST, ALT and GGT and BW were included as covariates in the models.

Differences in BW and enzyme activity levels at the start (day 1) and end of the experiment (day 21) between strains were analysed using generalized linear mixed models (PROC GLIMMIX). A log-normal distribution was used for the analysis of the enzyme activity levels and data were back transformed when necessary. Differences in the number of runs needed to reach the learning criterion during training and testing between strains were analysed using generalized linear mixed models (PROC GLIMMIX) with a negative binomial distribution. The activity levels of AST, ALT and GGT, and BW were included as covariates in the models. Initial values (day 1) were used as covariates while analysing the training phase, while final values (day 21) were used for the testing phase. In addition, the number of runs needed to reach the learning criterion during training was included as a covariate for the testing phase.

## Results

### Body weight and feeding behaviour

Body weight did not differ between LSL hens and UCD-003 hens at day 1 (1.73±0.043 kg *v*. 1.68±0.043 kg, *F*_1,46_=0.62, *P*=0.4336) and day 21 (1.72±0.039 kg *v*. 1.63±0.039 kg, *F*_1,44_=2.34, *P*=0.1334) of the experiment.

**Figure 2:**
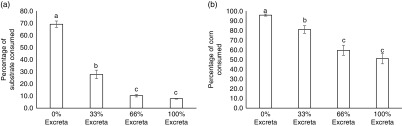
Feed preference of laying hens. (a) The average percentage of substrate consumed and (b) the average percentage of corn consumed from each treatment diet (0% excreta, 33% excreta, 66% excreta, and 100% excreta) for Lohmann LSL-Lite (LSL) and fatty liver haemorrhagic syndrome susceptible (UCD-003 strain) hens. The percentage consumed reflects the percentage of substrate or corn consumed from within each treatment. Different letters indicate significant differences (*P*<0.05).

While adjusting for BW, no diet-strain interaction was found for the percentage of substrate (*F*_3,138_=1.96, *P*=0.1231) and corn kernels (*F*_3,138_=0.70, *P*=0.5508) consumed. The feeding preference of laying hens for diets of different feed : excreta ratios are presented in Figure 2a. Both LSL and UCD-003 hens showed a clear decrease in the amount of substrate consumed as the percentage of excreta in the diet increased (*F*_3,138_=187.14, *P*<0.001). Approximately 70% of the substrate available in the container containing the 0% excreta diet was consumed, which was more than double of what was consumed from the container with 33% excreta (*t*_138_=9.37, *P*<0.001). Further reduction in the amount of substrate consumed was observed in the 66% excreta compared with 33% excreta diet (*t*_138_=4.77, *P*<0.001). However, the amounts consumed from diets with 66% excreta or 100% excreta were not statistically different (*t*_138_=2.36, *P*=0.0904). UCD-003 hens consumed similar amounts of substrate as LSL hens (30.4±1.60% *v*. 27.0±1.60%, *F*_1,46_=2.23, *P*=0.1422).

Similar results were observed for the consumption of corn kernels from each diet (Figure 2b). Hens showed a clear decrease in the percentage of corn kernels consumed as the percentage of excreta in the diet increased (*F*_3,138_=32.53, *P*<0.001). A lower percentage of corn was consumed from the 33% excreta compared with the 0% excreta diet (*t*_138_=4.47, *P*<0.001), and fewer corn kernels were consumed from the 66% than 33% excreta diet (*t*_138_=3.15, *P*=0.0106). The same percentage of corn was consumed from the 66% and 100% excreta diet (*t*_138_=1.14, *P*=0.6661). No difference in the percentage of corn consumed between birds from the UCD-003 strain (80.6±2.65%) and the LSL strain (74.3±3.20%) was found (*F*_1,46_=2.27, *P*=0.1386).

### Visual discrimination task and liver enzymes

One hen from the UCD-003 strain was moulting at the time of testing and was removed from the analysis. During visual discrimination learning (i.e. training), where a choice was given between two visually distinct arms of a Y-maze, all hens were capable of learning the task. Lohmann LSL-Lite hens took 26.0±3.01 runs to reach the learning criterion compared with UCD-003 hens which took 41.8±4.76 runs (*F*_1,8_=8.60, *P*=0.0189). During testing where birds had to recall the task after 21 days, no difference was found between LSL (9.4±2.00 runs) or UCD-003 hens (11.4±2.51 runs, *F*_1,7_=0.39, *P*=0.5523). A higher activity level of AST was associated with an increase in the number of runs needed to reach the learning criterion during testing (regression coefficient: 0.020±0.0076, *F*_1,7_=6.85, *P*=0.0345). The data for the activity levels of liver enzymes in laying hens are summarized in Table 1. No significant differences between LSL and UCD-003 hens were observed for the activity levels of ALT, AST and GGT, both at the start and the end of the experiment.

**Table 1:** Differences in plasma enzyme activity levels between Lohmann LSL-Lite (LSL) and fatty liver hemorrhagic syndrome susceptible (UCD-003) hens

	LSL	UCD-003		
	Mean±SE	Mean±SE	*F*-value	*P*-value
ALT (U/l)				
Initial (day 1)	4.8±1.31	2.2±0.65	*F*_1,8_=5.82	0.0878
Final (day 21)	3.5±0.76	2.1±0.50	*F*_1,8_=2.44	0.1567
AST (U/l)				
Initial (day 1)	163.6±12.98	176.7±15.37	*F*_1,8_=0.42	0.5332
Final (day 21)	141.4±9.27	143.3±10.29	*F*_1,8_=0.02	0.8975
GGT (U/l)				
Initial (day 1)	15.5±2.29	16.7±2.71	*F*_1,8_=0.12	0.7363
Final (day 21)	65.7±33.42	21.9±15.76	*F*_1,6_=1.55	0.2595

ALT=alanine aminotransferase; AST=aspartate aminotransferase; GGT=gamma-glutamyl transferase. The average activity levels expressed in units per litre (U/l) of ALT, AST and GGT measured in the blood of LSL (*n*=6) and UCD-003 (*n*=5) hens which completed the visual discrimination task at day 1 and day 21 of the experiment.

## Discussion

The goal of this study was to evaluate the effects of increasing excreta levels (0%, 33%, 66%, 100%) in diets on the amount consumed in a four-choice evaluation test. In addition, as hens naturally find feed by pecking at substrate with their beak (Savory *et al*., 1978), the motivation to search for and consume a luxury food reward (i.e. corn kernels) in these diets with increasing excreta levels as a digging substrate was investigated. Finally, we evaluated whether the birds’ preference is influenced by their physical/cognitive condition by using a commercial bird strain, predefined as metabolically healthy, and an experimental strain that is susceptible to liver damage, one of the leading non-infectious causes of mortality in commercial caged laying hens and obese backyard chickens (Trott *et al*., 2014).

Results showed that both LSL and UCD-003 hens prefer to feed and forage from diets without excreta, as indicated by the percentage of substrate and corn consumed from, in order from highest to lowest, 0%, 33% and 66% or 100% excreta diets, respectively. Familiarity of the feed must be accounted for, however, birds had time to familiarize themselves with the diets during habituation where birds saw, smelled and tasted the different excreta-feed diets. Features such as taste, smell and sight, texture and consistency during ingestion, followed by digestive tract distension and nutrient absorption in the post-ingestion period play a large role in feeding and foraging behaviour (Provenza, 1995), and likely determined the amount consumed over time. The 3-week testing period provided an appropriate period of time to establish a feed preference, when considering that pet food palatability testing typically lasts 4 to 6 days (Aldrich and Koppel, 2015).

Interestingly, despite enough feed being available in the 0% diet, hens still consumed substrate and corn from the diets mixed with excreta. Nearly 28% of the substrate available in the container with the 33% excreta diet was consumed by the birds, and even from the diets which consisted of 66% and 100% excreta some substrate was consumed, albeit at low levels (10% and 8%, respectively). The relatively higher amount of edible commercial feed in the 33% excreta diet with similar flavour and sensory characteristics of the 0% excreta diet compared with the 66% and 100% excreta diet, may have accounted for the proportion of substrate consumed from the 33% excreta diet. Additionally, the high water content of fresh excreta moistened the dry commercial feed to a porridge-like consistency which has been shown to increase feed intake in backyard poultry (Forbes, 2003). Another factor to consider is that birds likely were motivated to search for edible feed, but 33% of excreta in the diet was the price they were willing to pay in this search, explaining the relative preference for the 33% excreta diet over the 66% and 100% excreta diet. Relatively small amounts of substrate were consumed from the 66% and 100% excreta diets. This could in part be due to the birds’ foraging for corn in these diets, and as such, results should be interpreted with caution because feeding preference might be confounded with foraging for corn kernels in excreta. Similarly, Nicol *et al*. (2011) found that foraging decisions in laying hens are influenced by the environment, showing that hens demonstrated a pattern of preference for an environment with extra foraging opportunities, followed by a similar environment where a risk was perceived and finally an environment where only the risk was present. Our findings are also in agreement with Pokharel *et al*. (2018) who observed that hens spent more time foraging for feed on scratchpads soiled with excreta than on clean scratchpads in enriched cages. However, it must be noted that in both experiments birds were housed in an unnatural cage environment, where stimuli such as excreta may have been relatively salient and able to promote foraging behaviour. Nevertheless, this further underlines the importance of providing hens with appropriate foraging opportunities in farmed environments where animals are often presented with monotonous feed or litter substrates (Mellor, 2017).

Despite the presence of freely available feed (~134 g per day), birds consumed an average of 61.3 g of excreta diet per day (~37.2 g of the 33%, ~13.7 g of the 66%, and ~10.4 g of the 100% excreta diet). Similarly, Steffens and Menke (1964) reported from tracer studies with radioactive cobalt that chicks on litter consumed 5% to 24% of their excreta compared with 3% to 17% in chicks kept on wire flooring. Considering the average amount of excreta (~30.6 g per day) produced by a laying hen (Pokharel *et al*., 2018), birds ingested a considerable amount of excreta substrate from other laying hens. While the consumption of excreta in avian species has been associated with benefits in terms of nutritional recycling and reabsorption allowing birds to attain essential nutrients (Klasing, 2005; Golden *et al*., 2012), accurate values regarding the extent of excreta reingestion (i.e. coprophagy) in laying hens are lacking. It is suggested that cecal excreta are consumed by birds because it contains more bacteria and has high levels of protein, fat and vitamins (Klasing, 2005). It remains unclear why hens consume excreta from other hens, which should be further investigated from a physiological and psychological perspective, however this was beyond the scope of this experiment.

Hens showed a similar preference for unsoiled diet when foraging for corn, which was visually distinctive from the diet. In order from highest to lowest, most corn was consumed from 0%, 33% and 66% or 100% excreta diets. Corn kernels were mixed into the 134 g of diet in each container, meaning hens had to dig in increasing excreta levels (33%, 66% and 100% excreta) to find 81%, 60% and 51% of the corn kernels, respectively. To put this in perspective, hens only consumed 28%, 10% and 8% of these excreta diets, respectively, which indicates that hens did not encounter the corn kernels by chance but rather were specifically digging for the corn kernels, especially in the 66% and 100% excreta diets.

UCD-003 hens were used as a model for liver damage indicative of FLSH, however no indication of elevated activity levels of AST or ALT were found. Only GGT activity levels at the end of the experiment were outside of the normal range of 0 to 10 U/l (Harr, 2006), but this occurred in both strains. UCD-003 birds were maintained but not selected for liver damage over a period of 15 years at the research station, which might explain the lack of difference in plasma enzyme activities. Intriguingly, UCD-003 hens took more runs to successfully learn how to navigate a Y-maze compared with LSL hens. However, recalling the task after 21 days both LSL and UCD-003 hens performed equally well, needing only a few number of runs before reaching the learning criterion. While not statistically significant, the higher consumption of corn kernels by UCD-003 hens compared with LSL hens observed could indicate a slightly higher motivation for corn kernels, allowing UCD-003 hens to catch up with LSL hens’ performance during testing once they successfully learned the task hence explaining the equal number of runs. While a higher activity level of AST was associated with more runs to recall the task, the AST activity levels of the birds in our study did not reach outside of normal values (Hochleithner, 1994) and estimate values indicate this influence was not biologically relevant.

This study increased the understanding of feeding and foraging behaviour of laying hens. There is evident demonstration of a decreased preference for feeding and foraging from diets soiled with excreta. This is an important consideration for bird welfare as their preference for feeding and foraging in excreta-free diets is linked to their subjective experiences of their (un)naturalistic environment. However, it should be kept in mind that this preference may simply reflect the motivation at the time and can change depending on factors such as age, experience and conflicting needs. Nevertheless, we attempted to draw interferences on the strength of this preference by asking hens to forage for a luxury food reward (i.e. corn kernels) and, while hens foraged for corn in excreta diets, the preference pattern was consistent as shown by the lower consumption of a food reward in increasingly soiled diets. This could have implications for feed and litter management to allow hens to perform their natural foraging behaviour. Importantly, this study was the first to show that laying hens consume excreta from other laying hens as an initial investigation in the 1960s (Steffens and Menke, 1964). It should be taken into account that this study was conducted in individually cage-housed hens to allow for accurate measurement of consumption, which would be difficult in group and litter based housing systems. However, when accurate recording of excreta consumption is possible, future research under commercial conditions where hens are housed in social groups and in non-cage housing systems is needed to further understand how feeding preferences of laying hens are influenced. In addition, studies focussing on palatability, examining effects of different sensory qualities of excreta, whether birds prefer to consume ceca faeces or regular faeces, and possible nutritional effects of short- and long-term excreta consumption are needed. Furthermore, these results open a new field of research as the effects of consumption of excreta in farm animals kept in litter-based systems could have consequences for animal health and welfare that have not been previously considered.

## Conclusions

This study demonstrated a clear preference of laying hens for feeding and foraging on substrate free of excreta. However, considering the amount of excreta diets consumed, further studies are needed to understand the causes and consequences of excreta consumption on physiological and psychological functioning, and how this information can be used to allow adjustments in the management of foraging substrates in farmed birds.
